# Antimicrobial resistance of *Escherichia coli* from broilers in large-scale poultry farms in Shandong Province

**DOI:** 10.3389/fmicb.2025.1685522

**Published:** 2025-12-03

**Authors:** Xiaoxia Liu, Xiang Li, Jing Liu, Ruiying Chen, Rui Liu, Ruting Zhao, Jia Zhao, Jianwei Hao, Shuming Yang, Aiguo Luo, Ailiang Chen

**Affiliations:** 1 Department of Biological Science and Technology, Shanxi Center of Technology Innovation for Compound Condiment, Jinzhong University, Jinzhong, China; 2 Key Laboratory of Agro-Product Quality and Safety, Institute of Quality Standards and Testing Technology for Agro-Products, Chinese Academy of Agricultural Sciences, Beijing, China; 3 School of Food Science and Engineering, Shanxi Agricultural University, Jinzhong, China; 4 School of Investigation, People’s Public Security University of China, Beijing, China

**Keywords:** *Escherichia coli*, broiler, antibiotic resistance, multidrug resistance, factor

## Abstract

**Background:**

Antimicrobial resistance (AMR) is a global challenge affecting both healthcare and agricultural fields, as emphasized by the World Health Organization (WHO). Industrial poultry production plays a crucial role in the development and dissemination of AMR. A comprehensive understanding of the molecular mechanisms underlying AMR is imperative for developing effective control strategies.

**Materials and methods:**

This study aimed to identify factors influencing AMR in *Escherichia coli* from large-scale commercial broiler farms. Samples, including 371 anal swabs, 95 fecal swabs, and 122 feed-trough swabs, were collected from Cobb broilers at the four production stages (1, 15, 26, and 38 days of age) on typical farms in Shandong Province. From these specimens, 508 *E. coli* strains were isolated and characterized. Antimicrobial susceptibility was assessed using the Kirby–Bauer disk diffusion method against 15 commonly used antibiotics, and results were interpreted according to CLSI guidelines.

**Results:**

The resistance rates of the isolated strains varied between 24.41% and 95.47%. A total of 96.45% of the strains exhibited multidrug resistance, and 29 strains were resistant to all 15 antibiotics. The highest resistance was observed against penicillin (amoxicillin and ampicillin), followed by florfenicol, chloramphenicol, tetracycline, cefotaxime, and cefazolin. The lowest resistance was noted for ofloxacin and gentamicin. Drug resistance was most substantial at 15 days of age compared with that at 1, 26, and 38 days of age.

**Discussion:**

An analysis of the relationship between drug resistance and drug use showed that doxycycline significantly increased the resistance rate (68.40%, *p* < 0.05). Additionally, the drug resistance of bacteria isolated from fecal swabs was higher than that of bacteria from anal and feed-trough swabs. The results indicate that sample type, drug type, and age all influence *E. coli* drug resistance in poultry, with drug type having the greatest impact.

## 1 Introduction

Globally, the World Health Organization has identified antimicrobial resistance (AMR) as a major hazard to public health (Ajibola et al., 2025; Nduku et al., 2025; Donkor et al., 2024). The rise and proliferation of AMR pose significant challenges to global health, affecting humans, animals, and the environment (Ssemakadde et al., 2025; Ahmed et al., 2024; Melo et al., 2018; Badger et al., 2018; Eibach et al., 2018). Various factors contribute to the rising occurrence of AMR genes (Senawin et al., 2025; Pan et al., 2024; Bassitta et al., 2022). One of the primary drivers is the unregulated administration of antibiotics in lower-middle-income nations, where these medications are extensively used in both human and veterinary healthcare with inadequate oversight (Sunil et al., 2019; Hailat et al., 2023; Jimah et al., 2020; Opoku et al., 2020). The intricate dynamics of AMR, comprising interactions between humans, animals, and the environment, significantly influence the development and dissemination of resistance determinants (Lalak et al., 2016; Silbergeld et al., 2008).

Poultry has become an increasingly vital global source of nutrition, widely consumed yet frequently linked to outbreaks of foodborne illnesses. Harmful microorganisms may be transmitted to humans via direct exposure to poultry waste or ingestion of contaminated poultry derived products (Ajibola et al., 2025; Havelaar et al., 2022; Okpara et al., 2018; Herrero et al., 2013). The avian gastrointestinal tract serves as a reservoir for *Escherichia coli*, facilitating its potential transmission from birds to humans (Awawdeh et al., 2024; Wang et al., 2019; Shobrak and Abo-Amer, 2014). *E. coli* is a gram-negative bacterium that generally exists as a commensal organism in the digestive systems of animals, humans, and birds. However, some strains act as key pathogens, causing both intestinal and extraintestinal infections (Santos et al., 2020).

*Escherichia coli* shows a high potential for developing AMR relative to many other prevalent bacterial species. Thus, both non-pathogenic and disease, causing *E. coli* strains carry a wide range of AMR genes, thereby elevating the possibility of transferring resistance traits to humans and other living organisms (Ahmed et al., 2024; Watt et al., 2025; Chuppava et al., 2018; Badger et al., 2018; von Wintersdorff et al., 2016).

This study focused on Cobb broiler chickens in Shandong Province, with samples collected at various ages, separated, and analyzed for the presence of *E. coli*. These *E. coli* strains were evaluated for their sensitivity to a panel of 15 distinct antibiotics. Additionally, this study investigated variations in antibiotic resistance among chicken breeds at various ages. A comprehensive analysis of *E. coli* resistance in a large-scale poultry farming will offer a scientific framework for applying antimicrobial agents, thereby contributing to the containment of bacterial resistance. Moreover, the findings may facilitate optimizing breeding practices in poultry farms to reduce the burden of bacterial infections. This study aimed to elucidate the determinants influencing AMR in *E. coli* isolated from large-scale commercial broiler farms. By reducing the propagation of AMR and ensuring the safety and quality of poultry products, this study lays the foundation for further investigation into the resistance mechanisms of *E. coli*.

## 2 Materials and methods

### 2.1 Introduction to typical chicken farms and sample collection

This study was carried out on a broiler poultry farm in Shandong, China, spanning 30 acres with eight chicken houses, each containing 30,000 chickens (total 250,000 chickens). The feeding regimen for Cobb broiler chickens was planned for three stages: 1–15 days, 16–24 days, and 25–42 days within a 42-days feeding cycle. An all-in, all-out feeding strategy was employed, allowing the chickens *ad libitum* access to food and water. The farm adhered to rigorous disinfection and sterilization protocols before and after chicken occupancy. Vaccination against infectious bronchitis and Newcastle disease was administered via drinking water on day seven of the broiler feeding cycle. The farm’s medication protocol included tilmicosin and colistin on day 2 (treatment duration of 3–4 days), tilmicosin and colistin again on days 8 and 9, oxytetracycline on days 13 and 14, and amoxicillin and neomycin on days 28 and 29, each for 3–4 days.

Sample size determination and collection followed the methodological framework proposed by Apenteng et al. (2023), which emphasizes balancing statistical confidence, detection sensitivity, representativeness, and practical constraints in AMR surveillance. Following this principle, a total of 694 samples were initially collected from a representative large-scale Cobb broiler farm in Gaomi, Shandong Province. During transport, 106 samples were excluded due to physical damage, swab leakage, or inadequate sealing, all of which could lead to potential cross-contamination. After stringent quality control screening, 588 qualified samples were retained for analysis (Table 1). These comprised anal swabs, fecal swabs, and feed-trough swabs collected from broilers at four growth stages (1, 15, 26, and 38 days of age).

**TABLE 1:** Detailed sample collection (isolates) from a Cobb broiler farm in Shandong Province (strain) province.

Sample type	1-day-old		15-days-old		26-days-old		38-days-old		Total
	Southern region	Northern region	Southern region	Northern region	Southern region	Northern region	Southern region	Northern region	
Anal swab	116	32	57	51	45	30	15	25	371
Fecal swab	–	–	25	8	12	20	15	15	95
Feed-trough swab	18	25	10	10	10	16	15	18	122
Total	191		161		133		103		588

### 2.2 Isolation and purification of bacteria

The collected samples were shaken in a sterile physiological saline tube, and 1 mL was promptly added to the enrichment solution to isolate the bacteria. The tube was placed in a shaking incubator at 37 °C ± 1 °C for 18–24 h. In a sterile environment, a disposable inoculating loop was used to streak the cultured bacterial solution onto the appropriate selective medium, as outlined by Liu et al. (2022). After the culture period, suspicious single colonies observed on the plates were selected, inoculated onto nutrient agar plates, and preserved for further inspection. The isolation and identification processes adhered to relevant standards.

### 2.3 Molecular identification of test strains

Bacterial DNA was extracted by alkaline lysis following Clarridge’s (2004) methodology. The primer sequences were 27F (AGAGTTTGATCMTGGCTCAG) and 1492R (GGTTACCTTGTTACGACTT). The PCR amplification system was prepared in 20 μL (total volume), consisting of 2× PCR Master Mix (10 μL), 10 μmol/L of each of forward (F) and reverse (R) primers (1 μL), DNA template (20 ng), and ddH_2_O to adjust to the final volume. The thermal cycling conditions were as follows: an initial denaturation step at 94 °C for 4 min, followed by 30 cycles of denaturation at 94 °C for 40 s, annealing at 56 °C for 30 s, and elongation at 72 °C for 1 min, with a final elongation at 72 °C for 5 min. The amplified PCR products were submitted to Sangon Bioengineering (Shanghai) Co., Ltd., for sequencing. Sequence analysis was carried out using BLAST^1^. The Mega 6.0 software was used to conduct a comparative examination and construct phylogenetic trees as per the sequence similarity.

### 2.4 Drug susceptibility test of bacteria in typical chicken farms

The Kirby–Bauer (K–B) disk diffusion assay evaluated bacterial susceptibility to 15 distinct antibiotics. The interpretation of results and quality control procedures were conducted in line with the guidelines of the Clinical and Laboratory Standards Institute (CLSI) manual for antimicrobial susceptibility testing. Bacterial isolates were classified into three categories: susceptible (S), intermediate (I), or resistant (R) (Liu et al., 2022). The inhibition zone diameters were measured using Vernier calipers to determine the level of bacterial resistance to the tested antibiotics.

### 2.5 Data management and statistical analysis

All data were structured and processed via Microsoft Excel (Office 2013) and IBM SPSS Statistics 23. A logistic regression model was applied to assess the prevalence of *E. coli* resistance against multiple antimicrobial agents in broiler farming environments. Additionally, the impact of three factors–age, type of medication, and sampling location on *E. coli* resistance to each antibiotic was assessed using logistic regression analysis. The assigned values are presented in Supplementary Information Table SI 1 and were screened as per the odds ratios (OR values), 95% confidence intervals, and *p*-values to identify factors influencing *E. coli* resistance. A *p*-value of less than 0.05 or 0.001 was considered statistically significant. Key determinants influencing *E. coli* AMR were examined by evaluating the OR for each contributing factor. Factors with OR values deviating from one were regarded as particularly significant. Following the established criteria for multidrug resistant (MDR), extensively drug-resistant (XDR), and pan drug-resistant bacteria (PDR), *E. coli* isolates were categorized as fully susceptible, single-resistant (1), multi-resistant (2–4), or highly resistant (>4) to antibiotics (Magiorakos et al., 2012).

## 3 Results

### 3.1 Distribution of AMR rates in *E. coli* isolates from Cobb broiler farm

A total of 588 samples were processed for bacterial isolation, purification, and identification to obtain a comprehensive understanding of AMR in commercial poultry farms. As a result, 508 *E. coli* strains were isolated. The highest resistance levels among the isolated strains were observed against penicillin-like antibiotics, specifically amoxicillin (95.47%) and ampicillin (95.08%), as presented in Table 2. Notably, only 0.39% of isolates were intermediate to amoxicillin. Additionally, the investigated strains exhibited a resistance rate of 85.43% to florfenicol, widely used in clinical veterinary medicine.

**TABLE 2:** Distribution of overall drug resistance rate of *Escherichia coli* isolated from chicken in Cobb broiler farm.

Class	Antimicrobial	Sensitivity (S)	Intermediate (I)	Resistance (R)
		(*n*, %)	(*n*, %)	(*n*, %)
Penicillin	Amoxicillin	21 (4.13)	2 (0.39)	485 (95.47)
	Ampicillin	16 (3.15)	9 (1.77)	483 (95.08)
Chloramphenicol	Florfenicol	71 (13.98)	3 (0.59)	434 (85.43)
	Chloramphenicol	73 (14.37)	13 (2.56)	422 (83.07)
Sulfonamide synergist	Trimethoprim	112 (22.05)	5 (0.98)	391 (76.97)
Macrolide	Erythromycin	8 (1.57)	102 (20.08)	398 (78.35)
Tetracycline	Tetracycline	68 (13.39)	13 (2.56)	427 (84.06)
	Doxycycline	68 (13.39)	122 (24.02)	318 (62.60)
Cephalosporins	Cefotaxime	91 (17.91)	10 (1.97)	407 (80.12)
	Cefazolin	55 (10.83)	19 (3.74)	434 (85.43)
Quinolone	Ciprofloxacin	79 (15.55)	57 (11.22)	372 (73.23)
	Enrofloxacin	121 (23.82)	83 (16.34)	304 (59.84)
	Ofloxacin	242 (47.64)	142 (27.95)	124 (24.41)
Aminoglycoside	Gentamicin	230 (45.28)	91 (17.91)	187 (36.81)
	Streptomycin	93 (18.31)	22 (4.33)	393 (77.36)

### 3.2 Changes in drug-resistant bacteria during the broiler growth cycle

**FIGURE 1:**
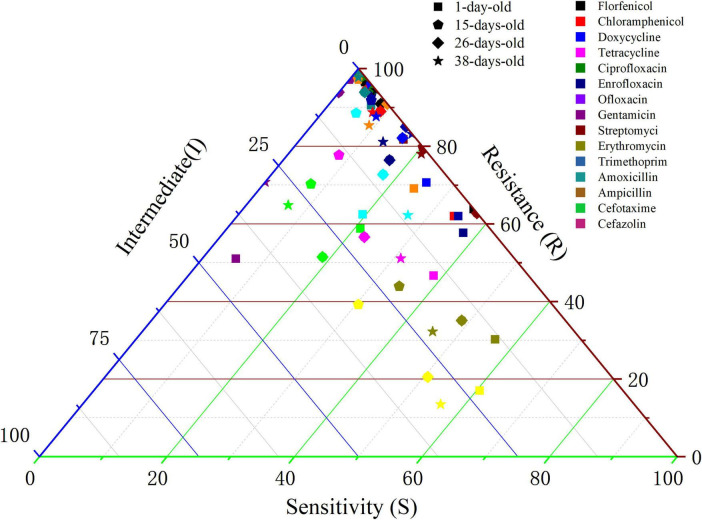
Results of antibiotic resistance of *Escherichia coli* isolated from Cobb broilers of different ages.

Sampling and analysis were conducted on Cobb broilers aged 1, 15, 26, and 38 days from large-scale commercial farms. Details of *E. coli* isolation are provided in Figure 1 and Supplementary Information Table SI 2. AMR was the lowest in isolates from 1-day-old chicks and peaked at 15 days of age. At this stage, isolates exhibited high resistance rates (approximately 90%) to multiple antibiotic classes, including penicillins (amoxicillin, ampicillin), chloramphenicol, florfenicol, macrolides (erythromycin), cephalosporins (cefotaxime, cefazolin), tetracyclines, and aminoglycosides (streptomycin). Notably, with the exception of ampicillin resistance in 1-day-old isolates (89.33%), resistance to penicillin antibiotics (amoxicillin and ampicillin) remained high across all sampling ages, consistently exceeding 90%.

In contrast, the sensitivity distribution of test strains isolated from Cobb broilers of varying ages to ofloxacin (a quinolone) revealed a clear trend, with an average resistance rate of 23.34%. Notably, the resistance rates of isolates on days 15 and 26 were higher than those observed on days 1 and 38, indicating a trend toward shifting drug resistance from sensitive to intermediate levels. Additionally, the resistance rates of strains isolated from different ages to chloramphenicol and tetracycline were comparable, with average resistance rates of 84.01% and 84.40%, respectively.

Resistance to florfenicol among isolates on days 15, 26, and 38 was notably high, exceeding 92%. In contrast, the resistance rate of isolates on day 1 was relatively low at 63.33%. Farm management records revealed that doxycycline was administered via drinking water on days 8 and 9, florfenicol on days 13 and 14, and amoxicillin and neomycin on days 28 and 29. Consequently, the resistance rates to doxycycline and florfenicol increased to 70.27% and 96.62%, respectively, by day 15, while the resistance rate to amoxicillin increased to 97.78% by day 38. These findings indicated that drug resistance was most pronounced on day 15, suggesting that the substantial use of antibiotics in the early stages contributed to increased resistance.

The influence of broiler age (1, 15, 26, and 38 days) on the AMR of *E. coli* was assessed using binary logistic regression, with isolates from 1-day-old chicks serving as the reference (Supplementary Information Table SI 3). The overall resistance was highest at 15 days of age, particularly to erythromycin (OR = 34.13, *p* < 0.001) and cefotaxime (*p* < 0.001). Resistance to chloramphenicol, erythromycin, and cefotaxime was consistently greater at 15, 26, and 38 days than at day 1 (*p* < 0.001). At 26 days, a notable increase in resistance to cefazolin (OR = 5.63, *p* < 0.001) was observed, accompanied by significant differences in susceptibility to trimethoprim, tetracycline, ciprofloxacin, and enrofloxacin (*p* < 0.05). By 38 days, resistance to amoxicillin, ampicillin, tetracycline, and cefazolin also remained significantly elevated (*p* < 0.05). Collectively, these findings indicate that *E. coli* resistance patterns fluctuate with age, with the highest overall resistance occurring at 15 days.

*Escherichia coli* isolated from Cobb broilers of different ages showed resistance to penicillin (amoxicillin and ampicillin), tetracyclines (tetracycline) and chloramphenicol-based fluorine compounds. Furthermore, the resistance rate analysis and binary logistic regression produced consistent results, suggesting a significant relationship between broiler age and *E. coli* resistance.

### 3.3 Relationship between drug-resistant bacteria and medication use

**FIGURE 2:**
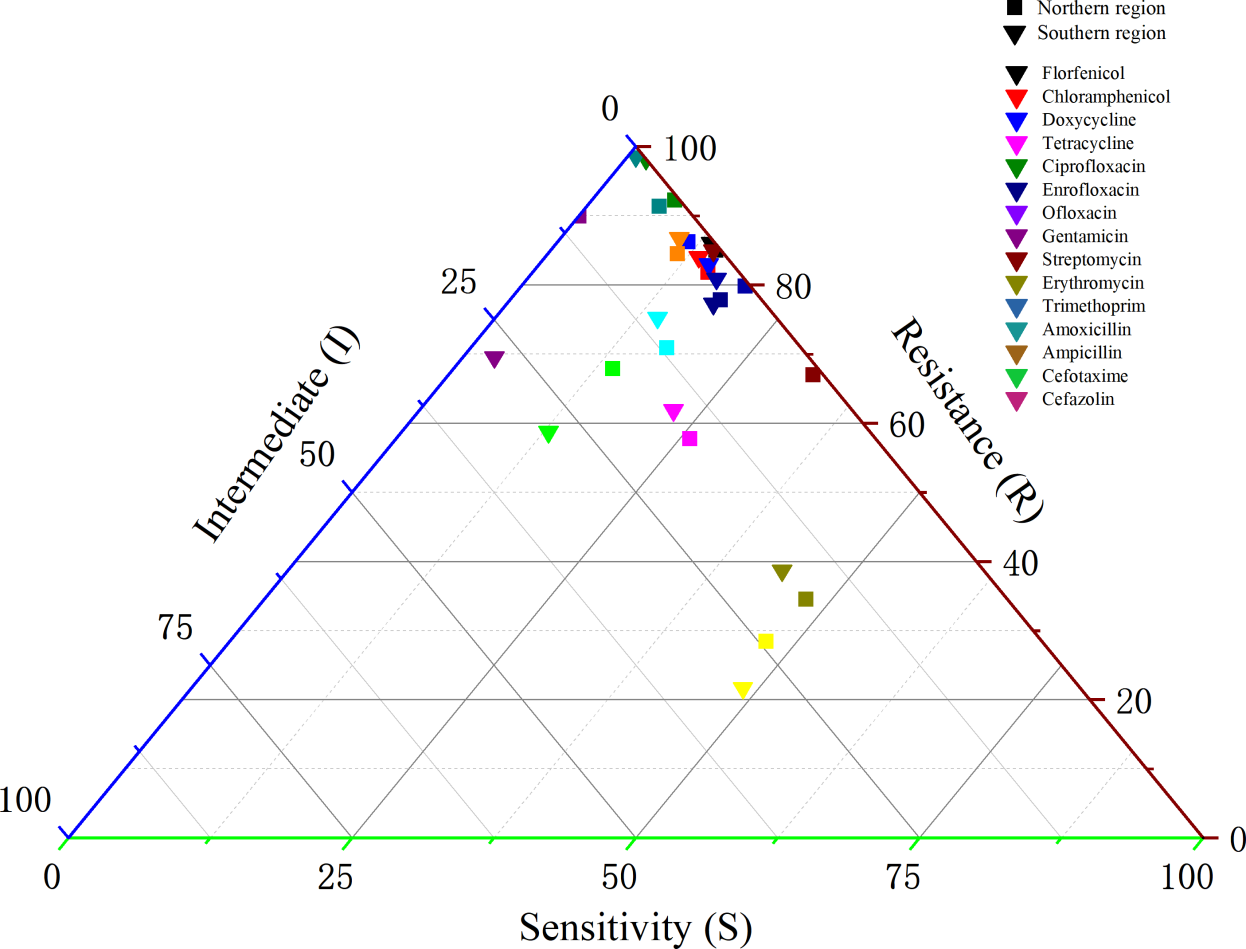
Effect of different ages on antibiotic susceptibility of *Escherichia coli* isolated from Cobb broilers.

Sampling and analysis were conducted at various locations within the southern (No. 1) and northern (No. 5) areas of a large-scale Cobb broiler farm. The *E. coli* strains isolated from chicken samples are presented in Figure 2 and Supplementary Information Table SI 4. Overall, the antibiotic resistance of isolates from the southern region was slightly higher than that of the isolates from the northern region. However, resistance to erythromycin, tetracycline, doxycycline, ofloxacin, and streptomycin was slightly higher among isolates from the northern region than in those from the southern region. In the southern area, the resistance rates to amoxicillin and ampicillin were 97.92% and 92.27%, respectively, while those to florfenicol and chloramphenicol were 85.76% and 84.03%, respectively. These resistance rates were similar to those observed in the northern areas.

The effect of various drug types on farms on the AMR of broiler derived *E. coli* was analyzed using binary logistic regression. The resistance profile of *E. coli* isolated from Cobb broilers in the southern area was used as a reference. The results of this analysis are presented in Supplementary Information Table SI 5. Compared with the southern region, *E. coli* isolates from the northern region showed significantly different resistance patterns to trimethoprim and erythromycin (*p* < 0.001), with ORs of 0.381 and 3.707, respectively. This indicated that the resistance of *E. coli* to trimethoprim and erythromycin in the northern region was particularly pronounced. However, substantial variations in resistance were identified for amoxicillin, ampicillin, and doxycycline (*p* < 0.05), with corresponding OR values of 0.254, 0.225, and 1.531, respectively. These findings indicate that *E. coli* isolates from the southern region showed remarkable resistance to amoxicillin and ampicillin. Moreover, the observed resistance to these β-lactam antibiotics and doxycycline was substantial and corresponded with the documented antimicrobial administration practices during farm visits.

### 3.4 Relationship between sampling type and drug-resistant bacteria

**FIGURE 3:**
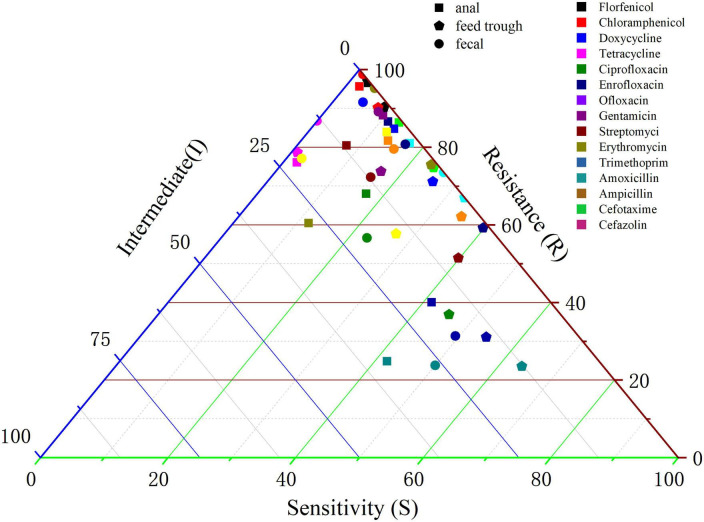
Effect of different ages on antibiotic resistance in *Escherichia coli* isolated from Cobb broilers.

Sampling and analyses were conducted on various sample types (anal swabs, fecal swabs, and feed-trough swabs) of a large-scale Cobb broiler farm in Shandong. The antimicrobial susceptibility findings for the isolated *E. coli* strains are detailed in Supplementary Information Table SI 6 and illustrated in Figure 3. Statistical analysis indicated that the resistance of the test strains isolated from fecal swabs to antibiotics was greater than that observed in isolates from anal and feed-trough swabs. Notably, the resistance rates of isolates from fecal swabs to ampicillin, amoxicillin, florfenicol, chloramphenicol, and tetracycline exceeded 91%, suggesting a relatively high resistance level in fecal swabs.

Binary logistic regression was employed to assess the effect of different samples (anal, fecal, and trough swabs) on the AMR of *E. coli*. For this analysis, *E. coli* isolates obtained from anal swabs in Cobb broiler farms were used as the reference group. The findings are summarized in Supplementary Information Table SI 7. As shown in the table, resistance patterns in *E. coli* isolates from trough swabs differed substantially from those in anal swabs for all tested antibiotics except for erythromycin, gentamicin, and doxycycline (*p* < 0.05). Furthermore, the resistance of the tested bacteria to cefotaxime, ciprofloxacin, and streptomycin was significantly different (*p* < 0.001). Compared with those from anal swabs, *E. coli* isolated from fecal swabs of chickens demonstrated marked differences in resistance to erythromycin, doxycycline, tetracycline, and florfenicol, with ORs of 2.057, 2.222, 3.717, and 3.126, respectively.

### 3.5 Distribution of multidrug-resistant chicken-derived *E. coli* isolates in broiler farms

**FIGURE 4:**
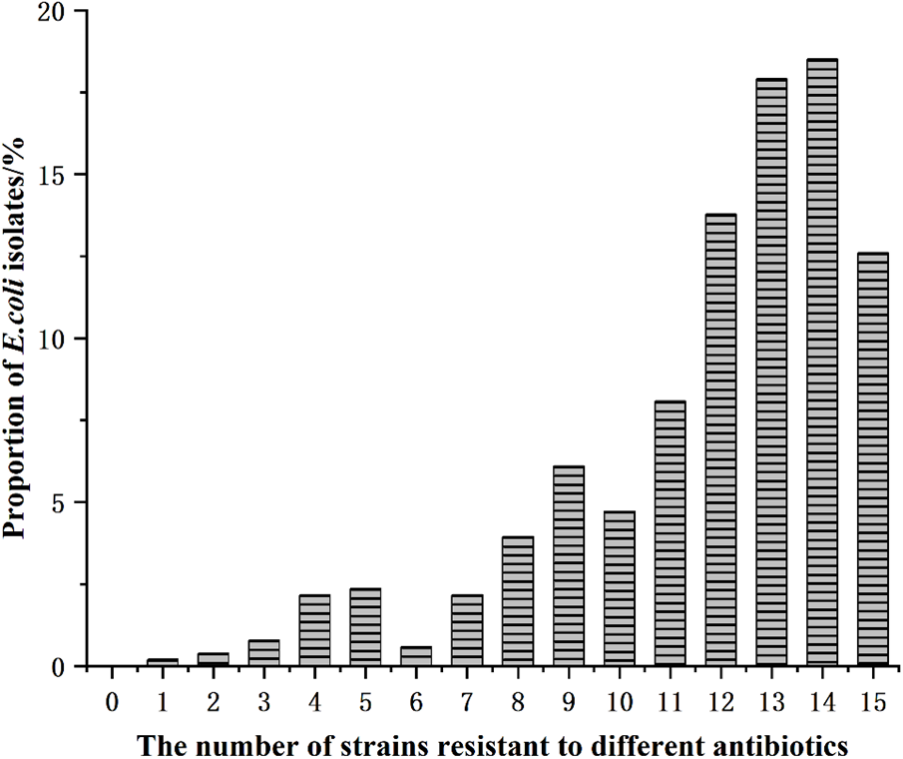
The number of strains resistant to different antibiotics.

Antibiotic resistance profiles of 588 *E. coli* isolates obtained from Cobb broiler farms were analyzed, and the distribution of resistant isolates is summarized in Figure 4 and Table 3. The number of resistant *E. coli* isolates varied substantially, with the largest proportion (18.50%) showing resistance to 13 antibiotics. Resistance to fewer than three antibiotics was rare (3.55%), whereas 2.36% and 2.17% of the isolates were resistant to four and six antibiotics, respectively. A high level of resistance was predominantly observed among isolates resistant to 11–13 antibiotics, and 29 isolates exhibited resistance to all tested antibiotics. These findings highlight the widespread occurrence and the alarming extent of antibiotic resistance among *E. coli* isolates throughout the broiler growth cycle.

**TABLE 3:** Distribution of *Escherichia coli* strains resistant to different antibiotics in Cobb broiler farms.

Number of strains resistant	(*n*, %)	Number of strains resistant	(*n*, %)
0	1 (0.20)	8	31 (6.10)
1	2 (0.39)	9	24 (4.72)
2	4 (0.79)	10	41 (8.07)
3	11 (2.17)	11	70 (13.78)
4	12 (2.36)	12	91 (17.91)
5	3 (0.59)	13	94 (18.50)
6	11 (2.17)	14	64 (12.60)
7	20 (3.94)	15	29 (5.71)

#### 3.5.1 Distribution of resistant *E. coli* isolates from Cobb broiler farms at different ages

**FIGURE 5:**
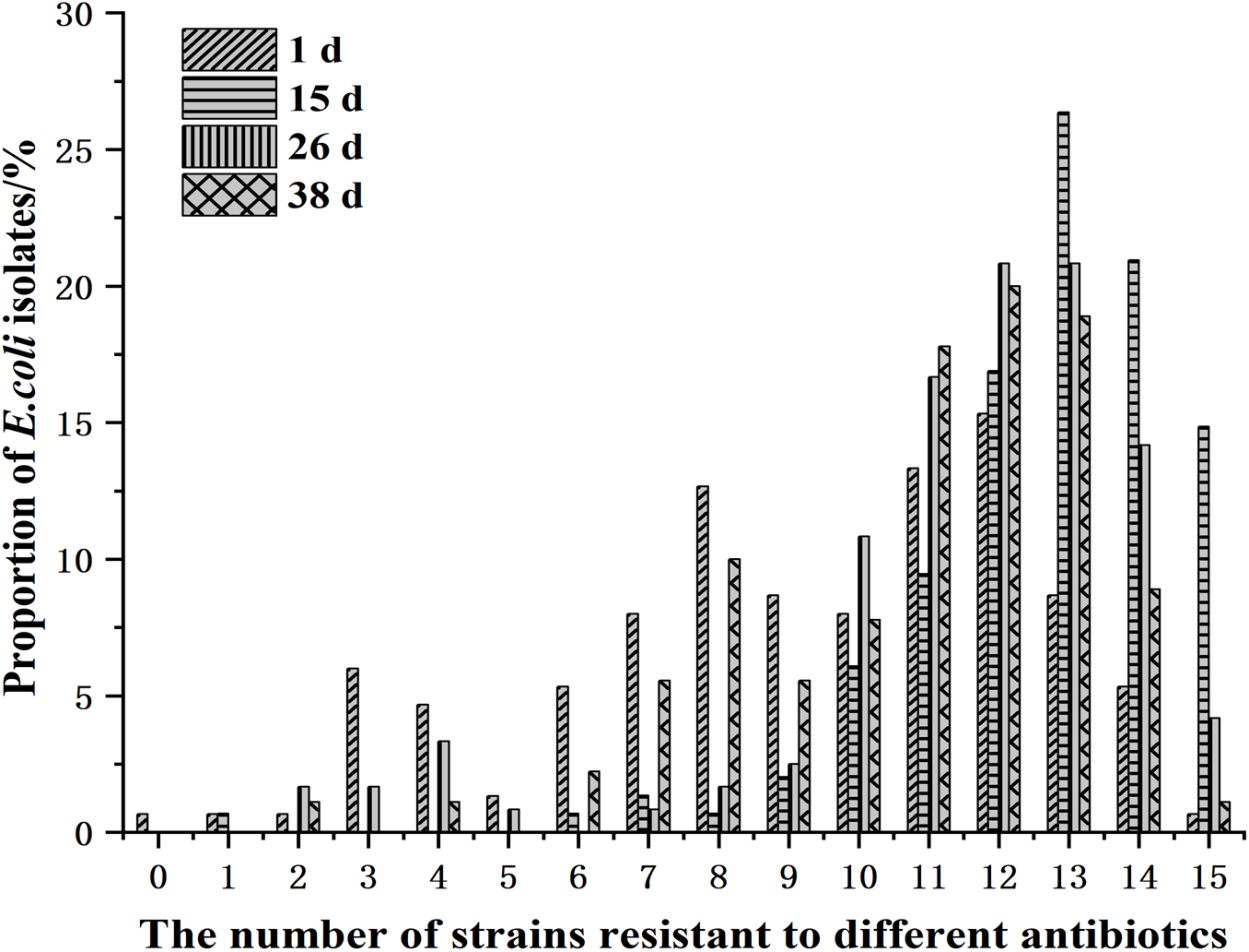
Distribution of *E. coli* strains resistant to different antibiotics at different ages.

Statistical analysis of the number of resistant *E. coli* isolates from Cobb broiler farms across different sampling days is presented in Figure 5 and Supplementary Information Table SI 8. At 1 day of age, the range of resistant *E. coli* strains was 1–15 antibiotics, predominantly demonstrating resistance to 11–12 antibiotics. By 15 days of age, the range increased to 6–15 antibiotics, with resistance mainly observed in 13–14 antibiotics. At 26 days of age, the range was 2–15 antibiotics (excluding six classes), with resistance primarily concentrated in 12–13 antibiotics.

Additionally, isolates from day-old chicks exhibited resistance to 2–15 antibiotics (excluding five classes), again concentrated in 12–13 antibiotic classes. At the fourth growth stage, the number of resistant *E. coli* isolates was primarily associated with 12–13 antibiotic classes. Considering the entire growth cycle of broiler chickens, the extent of resistance among *E. coli* isolates was most pronounced at 15 days of age.

#### 3.5.2 Distribution of resistant *E. coli* isolates from Cobb broiler farms under different medication types

**FIGURE 6:**
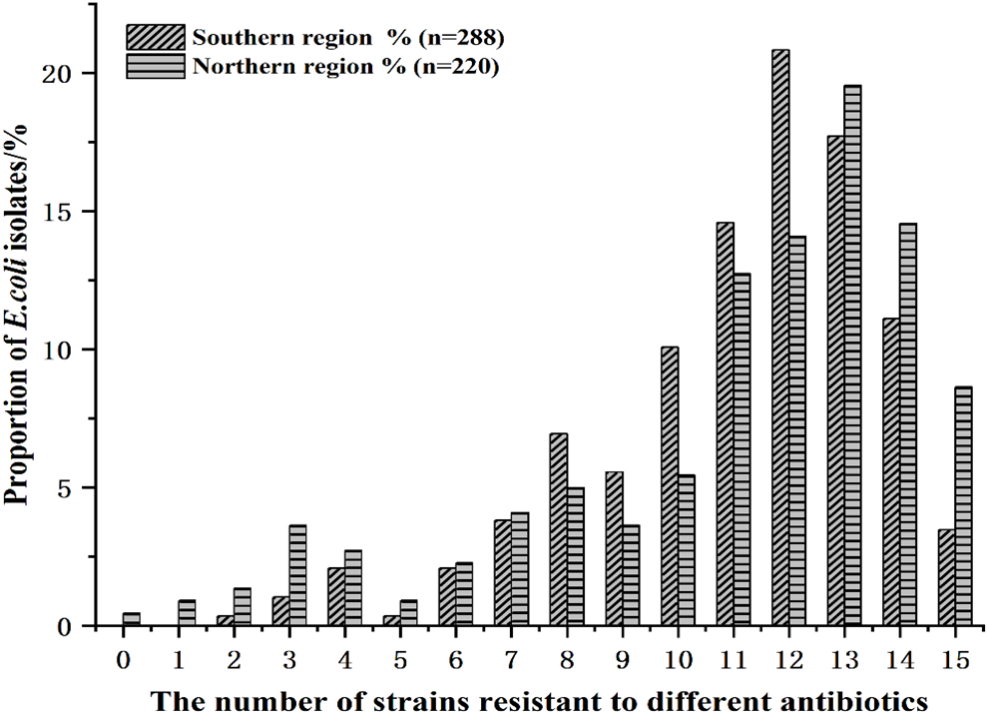
Distribution of antibiotic resistant *E. coli* isolates from Southern and Northern regions of Cobb broilers.

Statistical analyses of the number of resistant *E. coli* isolates and the corresponding antibiotics by medication type are presented in Figure 6 and Supplementary Information Table SI 9. In the southern region, the resistant *E. coli* strains were mainly distributed across 12–13 antibiotics, whereas in the northern region, they were concentrated in 13–14 antibiotics.

#### 3.5.3 Distribution of resistant *E. coli* isolates from Cobb broilers by sample type

**FIGURE 7:**
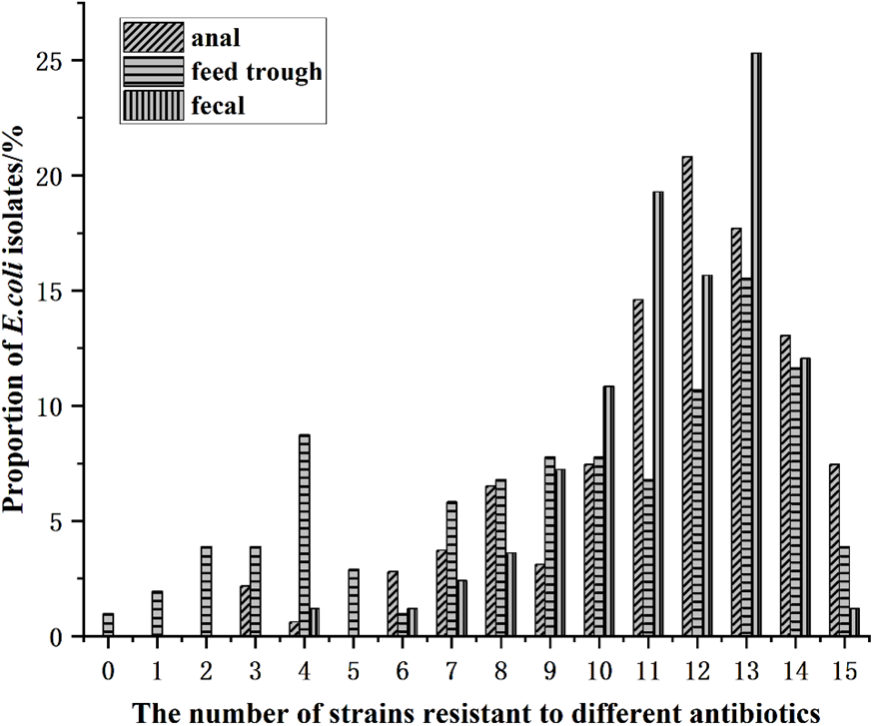
Distribution of antibiotic resistant *E. coli* isolates from different parts of Cobb broilers.

Statistical analyses of the number of resistant *E. coli* isolates and the corresponding antibiotics by sample type (anal swabs, feed-trough swabs, and fecal swabs) are presented in Figure 7 and Supplementary Information Table SI 10. Resistant *E. coli* strains isolated from anal swabs were predominantly resistant to 12–13 antibiotics, whereas those from feed-trough swabs exhibited resistance to 13–14 antibiotics. Fecal swab isolates were resistant to 13 antibiotics. Notably, the occurrence of extensively drug-resistant *E. coli* originating from chickens was particularly high in anal swab samples.

## 4 Discussion

Antimicrobial agents are essential in controlling various infections (Islam et al., 2024; Arbab et al., 2022). They target diverse pathogens, such as bacteria (antibiotics), fungi (antifungals), viruses (antivirals), and parasites (including antimalarials). However, the increasing drug resistance of *E. coli* in livestock and poultry production has complicated the understanding of underlying resistance mechanisms. This complexity, combined with the increasing dosages required to treat animal diseases, has resulted in suboptimal treatment outcomes and significant challenges in both prevention and control. Consequently, drug resistance poses major difficulties for the management of bacterial diseases.

Our study documents a pervasive AMR burden among broiler derived *E. coli* on a large-scale poultry farm. Resistance was highest for β-lactams, chloramphenicol, tetracycline, and early/third-generation cephalosporins, whereas ofloxacin and gentamicin showed comparatively lower resistance. Multidrug resistance (MDR) was widespread among isolates. These patterns are consistent with intensive poultry production and align with farm-level treatment practices reported during the production cycle (Table 2). To contextualize these findings, we compare our data with prior research and farm-level medication records.

At our site, 508 *E. coli* isolates were recovered from typical broiler farms in Shandong Province. Consistent with Table 2, the highest resistance levels were observed for penicillin derivatives, specifically amoxicillin (95.47%) and ampicillin (95.08%). Resistance exceeded 80% for florfenicol (85.43%), chloramphenicol, tetracycline (84.06%), cefotaxime (80.12%), and cefazolin (85.43%). Although ofloxacin (24.41%) and gentamicin (36.81%) showed the lowest resistance rates, both remained above 20%. Additionally, resistance rates above 50% were observed for sulfamethoxazole, erythromycin, streptomycin, doxycycline, ciprofloxacin, and enrofloxacin. Notably, 96.45% of the strains displayed MDR. The findings of this study align with those of previous research (de Jong et al., 2009; Lee, 2009). Nationally, cefotaxime resistance in poultry and swine *E. coli* increased from 25.60% (2008) to 46.91% (2012) and then declined to 35.08% (Zhang et al., 2015). Compared with the resistance levels reported in the US and the European Union, the prevalence of resistance to third-generation cephalosporins in Chinese poultry and swine farms remains substantially higher. A study by Tadesse et al. (2012) indicated that *E. coli* isolates derived from both human and animal sources showed a cefotaxime resistance rate of <22%, suggesting a lower prevalence of resistance in their findings relative to the current study.

Variation in AMR patterns among chicken breeds and age groups underscores the complex dynamics of AMR in poultry production. Although this study focused specifically on Cobb broilers, other strains (e.g., Ross and Hubbard) may display different resistance profiles owing to genetic differences, management practices, and immune capacity (Chuppava et al., 2018; Awawdeh et al., 2024). Our results indicated that isolates from 15-days-old broilers exhibited the highest levels of resistance, suggesting a close association between frequent antimicrobial use during the early life stage and a marked increase in *E. coli* resistance. During the chick stage, the immune system is immature, and birds are highly susceptible to environmental microbes. Under the high stocking densities typical of intensive production, producers often rely on antimicrobials to prevent disease, reduce losses, and maximize economic returns. Consistent with our findings, Zhang et al. (2017) reported a pronounced increase in resistance during early growth, particularly at the outset of intensive rearing, attributable to frequent antimicrobial use and immune immaturity. Zhou et al. (2024) similarly observed that *E. coli* isolated from white-feathered broilers maintained persistently high resistance to most antibiotics throughout the rearing cycle, with tetracycline resistance showing only minor fluctuations after 21 days of age and no significant decline.

Fenollar-Penadés et al. (2024) reported that, during the laying-hen rearing period, the prevalence of MDR in *E. coli* and resistance levels to some individual antimicrobials tended to decrease with increasing bird age; however, resistance remained widespread overall. Major resistance determinants (e.g., blaTEM, blaSHV, qnrB) were detected consistently across all stages, indicating persistence and co-occurrence of resistance genes. Moreover, Brower et al. (2017) characterized the antibiotic resistance landscape of *E. coli* within poultry production systems in Punjab, India, finding substantially higher resistance in broilers than in layers. This disparity was closely associated with intensive antimicrobial use and the short production cycles typical of broiler systems. The study further demonstrated that breed specific resistance patterns reflect differences in antimicrobial management practices and underscored the need for stricter antibiotic stewardship in broiler production. These breed and age associated differences underscore the necessity for tailored antimicrobial management strategies: changes in AMR patterns should be interpreted with regard to production type, growth stage, and genetic background.

The administration of antimicrobial agents is closely related to the emergence of bacterial resistance. A study by Zhang et al. (2017) examined the patterns of *E. coli* antibiotic resistance across seven provinces in China, including Sichuan, Shanghai, Shandong, Guangdong, Liaoning, Henan, and Inner Mongolia, from 2008 to 2015. The study found that 7,568 *E. coli* strains were isolated from chicken feces, with an MDR rate of 89.20% (6,571 out of 7,568). Notably, the high resistance rates (exceeding 86%) of chicken-derived *E. coli* to ampicillin, tetracycline, and sulfonamide suggest the prevalence of resistance to these three antibiotics on chicken farms. This phenomenon may be attributed to the frequent use of antibiotic classes (tetracyclines, sulfonamides, and penicillin) in agriculture. Antibiotics such as colistin, doxycycline, florfenicol, amoxicillin, and neomycin were used in their study. Their results indicated a significant increase in the resistance rate following antibiotic use. Speksnijder et al. (2015) reached a similar conclusion. Meanwhile, Lee (2009) reported that young poultry exhibited elevated resistance to ampicillin and tetracycline, a pattern that may be closely associated with the prophylactic use of antimicrobials during critical developmental stages.

Chloramphenicol has historically been used in multiple sectors but has been restricted in food animals in many regions due to safety concern (Sathya et al., 2020; Wang et al., 2021). To strengthen the regulation of veterinary drug residues, the Ministry of Agriculture of the People’s Republic of China (MOA) issued Circular No. 235 in December 2002, titled “Maximum Residue Limits of Veterinary Drugs in Animal Foods,” which explicitly banned chloramphenicol in animal-derived food products. Similarly, due to concerns over bacterial resistance and the toxic effects of chloramphenicol, several countries and areas i.e., Canada, the US, the European Union, Japan, Australia, and Brazil have established regulatory measures to restrict its use (Barreto et al., 2016). Despite its prohibition in veterinary medicine, the present study observed a resistance level of 83.07%, which closely aligns with the resistance to florfenicol, a structurally related antimicrobial agent. These findings indicate the potential for resistance transmission among bacterial species.

## 5 Strengths and limitations

Strengths include stage-stratified sampling at four production ages and across three sample types (anal swabs, fecal swabs, and feed-trough swabs), the relatively large number of isolates (*n* = 508), and the use of standardized CLSI-interpreted K–B disk diffusion testing across 15 agents, combined with farm-level treatment records. Limitations include the single-farm scope, which may limit the generalizability of the findings; the absence of genotypic characterization (e.g., extended-spectrum beta-lactamases, plasmid-mediated quinolone resistance genes, and aminoglycoside-modifying enzymes) to identify the underlying resistance mechanisms; and potential within-house clustering or unmeasured management confounders. The observational design precludes causal inference, and phenotypic resistance patterns may not fully capture transmission dynamics.

Future studies should broaden the sampling scope across multiple farms and integrate molecular or whole-genome analyses to determine the genetic relationships among animal, environmental, and human isolates. Such approaches would enhance understanding of antimicrobial transmission pathways and support targeted interventions aimed at mitigating resistance dissemination.

## 6 Conclusion

This study demonstrated that *E. coli* isolates from broiler farms in Shandong exhibited widespread MDR, particularly against penicillin and chloramphenicol. Resistance profiles varied across the growth cycle, with mid-growth broilers showing the highest resistance levels. The use of doxycycline was associated with elevated resistance, highlighting the selective pressure exerted by antimicrobial exposure. Moreover, fecal isolates showed higher resistance than those from anal or feed-trough samples, indicating that sampling type can influence observed AMR patterns. Collectively, these findings suggest that broiler growth stage, sampling type, and medication practices are key determinants shaping the resistance dynamics of broiler derived *E. coli*. Strengthening prudent antimicrobial use and maintaining continuous surveillance throughout the growth cycle are essential for mitigating the spread of resistance within intensive poultry production systems.

## Data Availability

The original contributions presented in this study are included in this article/Supplementary material, further inquiries can be directed to the corresponding authors.

## Footnotes

1
http://www.ncbi.nlm.nih.gov/BLAST
