# Supplementary material for: Antimicrobial resistance of Escherichia coli from broilers in large-scale poultry farms in Shandong Province
Source: Front Microbiol. 2025 Dec 3;16:1685522. doi: 10.3389/fmicb.2025.1685522 (PMC12708605; doi:10.3389/fmicb.2025.1685522)
Supplement: Supplementary file 1 [file Table_1.docx]

**Table SI 1** Assignment description of binary logistic regression analysis of *Escherichia coli* resistance in typical chicken farm

| Factor | | Assignment description | |
| --- | --- | --- | --- |
| Different drug types | | | Southern region = 0，Northern region = 1 |
| Different ages | 1-day-old = 0，15-days-old = 1，26-days-old = 2，38-days-old = 3 | | |
| Sample type | | | Anal swab = 0，feces = 1，feed trough = 2 |
| Resistance | | Sensitivity = 0, Resistance = 1 | |
| Factor | | Assignment description | |

**Table SI 2** Resistance of *Escherichia coli* isolated from Cobb broilers of different ages to antibiotics

| Antimicrobial |  |  | Number (%) of strains | | | Average resistance  (%) |
| --- | --- | --- | --- | --- | --- | --- |
|  | 1-day-old  % (n = 150) | | 15-days-old  % (n = 148) | 26-days-old  % (n = 120) | 38-days-old  % (n = 90) |  |
| Amoxicillin | 136 (90.67) | | 146 (98.65) | 115 (95.83) | 88 (97.78) | 95.73 |
| Ampicillin | 134 (89.33) | | 147 (99.32) | 114 (95.00) | 88 (97.78) | 95.36 |
| Florfenicol | 95 (63.33) | | 143 (96.62) | 111 (92.50) | 85 (94.44) | 86.72 |
| Chloramphenicol | 93 (62.00) | | 140 (94.59) | 110 (91.67) | 79 (87.78) | 84.01 |
| Trimethoprim | 121 (80.67) | | 118 (79.73) | 81 (67.50) | 71 (78.89) | 76.70 |
| Erythromycin | 77 (51.33) | | 144 (97.30) | 114 (95.00) | 63 (70.00) | 78.41 |
| Doxycycline | 87 (58.00) | | 104 (70.27) | 68 (56.67) | 59 (65.56) | 62.62 |
| Tetracycline | 106 (70.67) | | 141 (95.27) | 102 (85.00) | 78 (86.67) | 84.40 |
| Cefotaxime | 93 (62.00) | | 135 (91.22) | 105 (87.50) | 74 (82.22) | 80.73 |
| Cefazolin | 103 (68.67) | | 144 (97.30) | 111 (92.50) | 76 (84.44) | 85.73 |
| Ciprofloxacin | 93 (62.00) | | 131 (88.51) | 92 (76.67) | 56 (62.22) | 72.35 |
| Enrofloxacin | 69 (46.00) | | 115 (77.70) | 74 (61.67) | 46 (51.11) | 59.12 |
| Ofloxacin | 25 (16.67) | | 58 (39.19) | 29 (24.17) | 12 (13.33) | 23.34 |
| Gentamicin | 45 (30.00) | | 65 (43.92) | 48 (40.00) | 29 (32.22) | 36.54 |
| Streptomycin | 86 (57.33) | | 138 (93.24) | 96 (80.00) | 73 (81.11) | 77.92 |

**Table SI 3** Analysis of antibiotic resistance of *Escherichia coli* isolated from Cobb broilers of different ages by logical regression method

|  | OR value (95% CI) | | | | | |
| --- | --- | --- | --- | --- | --- | --- |
| Antimicrobial | 15 d | | 26 d | 38 d | | |
| Amoxicillin | 7.515 (1.677–33.675)* | 2.368 (0.828–6.772) | | | | 4.529 (1.005–20.415)* |
| Ampicillin | 17.552 (2.296– 134.153)* | 2.269 (0.859– 5.990) | | | | 5.254 (1.179– 23.413)* |
| Florfenicol | 0.102 (0.039–0.266)** | 1.682 (0.473–5.981) | | | | 0.725 (0.235–2.244) |
| Chloramphenicol | 10.726 (4.892–23.517)** | 6.742 (3.260–13.941)** | | | | 4.402 (2.160–8.969)** |
| Trimethoprim | 0.943 (0.533–1.667) | 0.498 (0.285–0.869)* | | | | 0.896 (0.468–1.713) |
| Erythromycin | 34.130 (12.017–96.930)** | 18.013 (7.463–43.478)** | | | | 2.212 (1.273–3.846)** |
| Tetracycline | 8.361 (3.623–19.298)** | 2.352 (1.275–4.338)* | | | | 2.698 (1.337–5.445)* |
| Doxycycline | 1.712 (1.060–2.763)* | 0.947 (0.583–1.538) | | | | 1.378 (0.801–2.371) |
| Cefotaxime | 6.365 (3.297–12.287)** | 4.290 (2.277–8.084)** | | | | 2.835 (1.505–5.338)** |
| Cefazolin | 16.427 (5.739–47.023)** | 5.628 (2.627–12.057)** | | | 2.477 (1.272–4.823)* | |
| Ciprofloxacin | 4.723 (2.583–8.634)** | 2.014 (1.178–3.443)* | | | 1.009 (0.589–1.730) | |
| Enrofloxacin | 4.091 (2.474–6.764)** | 1.888 (1.159–3.078)* | | | 1.227 (0.727–2.071) | |
| Ofloxacin | 3.222 (1.875–5.538)** | 1.593 (0.875–2.901) | | | 0.769 (0.365–1.619) | |
| Gentamicin | 1.827 (1.134–2.944)* | 1.556 (0.938–2.578) | | | 1.109 (0.632–1.949) | |
| Streptomycin | 10.270 (5.005–21.073) | 2.977 (1.714–5.170) | | | 3.196 (1.721–5.935) | |

Note: The OR denotes the probability of *E. coli* resistance in swabs on days 15, 26, and 38 relative to day 1, 95 % CI, *p < 0.05，**p < 0.001.

**Table SI 4** AMR patterns of *Escherichia coli* isolates from Southern and Northern regions at Cobb broiler farms

| Antimicrobial | Number of strains (%) | | | | |
| --- | --- | --- | --- | --- | --- |
|  | Southern region  (No. 1) | % (n = 288) | Northern region  (No. 5) | % (n = 220) | Average resistance  (%) |
| Amoxicillin | 282 | 97.92 | 203 | 92.27 | 95.10 |
| Ampicillin | 282 | 97.92 | 201 | 91.36 | 94.64 |
| Florfenicol | 247 | 85.76 | 187 | 85.00 | 85.38 |
| Chloramphenicol | 242 | 84.03 | 180 | 81.82 | 82.93 |
| Trimethoprim | 243 | 84.38 | 148 | 67.27 | 75.83 |
| Erythromycin | 201 | 69.79 | 197 | 89.55 | 79.67 |
| Tetracycline | 239 | 82.99 | 188 | 85.45 | 84.22 |
| Doxycycline | 197 | 68.40 | 139 | 63.18 | 65.79 |
| Cefotaxime | 233 | 80.9 | 174 | 79.09 | 80.00 |
| Cefazolin | 249 | 86.46 | 185 | 84.09 | 85.28 |
| Ciprofloxacin | 216 | 75.00 | 156 | 70.91 | 72.96 |
| Enrofloxacin | 177 | 61.46 | 127 | 57.73 | 59.60 |
| Ofloxacin | 62 | 21.53 | 62 | 28.18 | 24.86 |
| Gentamicin | 111 | 38.54 | 76 | 34.55 | 36.55 |
| Streptomycin | 221 | 76.74 | 172 | 78.18 | 77.46 |

**Table SI 5** Analysis of AMR patterns of *Escherichia coli* isolates from Southern and Northern regions at Cobb broiler farms

|  | Northern region (No. 5) | | |
| --- | --- | --- | --- |
| Antimicrobial | OR value | 95% CI (confidence interval) | P |
| Amoxicillin | 0.254 | 0.098–0.656 | 0.005* |
| Ampicillin | 0.225 | 0.088–0.574 | 0.002* |
| Florfenicol | 0.941 | 0.573–1.545 | 0.809 |
| Chloramphenicol | 0.855 | 0.537–1.363 | 0.511 |
| Trimethoprim | 0.381 | 0.249–0.582 | 0.000** |
| Erythromycin | 3.707 | 2.249–6.111 | 0.000** |
| Tetracycline | 1.204 | 0.742–1.956 | 0.452 |
| Doxycycline | 1.531 | 1.059–2.211 | 0.023* |
| Cefotaxime | 0.893 | 0.576–1.384 | 0.612 |
| Cefazolin | 0.828 | 0.505–1.357 | 0.454 |
| Ciprofloxacin | 0.813 | 0.548–1.206 | 0.303 |
| Enrofloxacin | 0.856 | 0.599–1.225 | 0.395 |
| Ofloxacin | 1.430 | 0.953–2.148 | 0.084 |
| Gentamicin | 0.842 | 0.584–1.213 | 0.355 |
| Streptomycin | 1.086 | 0.713–1.655 | 0.700 |

Note: The OR denotes the probability of *E. coli* resistance in swabs from the northern region relative to the southern region, 95 % CI, *p < 0.05，**p < 0.001

**Table SI 6** AMR patterns of *Escherichia coli* isolates from chickens collected from different parts of Cobb broilers

|  | Number (%) of drug-resistant strains | | | | | | | |
| --- | --- | --- | --- | --- | --- | --- | --- | --- |
| Antimicrobial | Anal | % (R/322) | Feces | % (R/83) | | Feed trough | % (R/103) | Average resistance (%) |
| Amoxicillin | 311 | 96.58 | 81 | 97.59 | | 93 | 90.29 | 94.82 |
| Ampicillin | 309 | 95.96 | 82 | 98.80 | | 92 | 89.32 | 94.69 |
| Florfenicol | 278 | 86.34 | 79 | 95.18 | | 77 | 74.76 | 85.42 |
| Chloramphenicol | 272 | 84.47 | 76 | 91.57 | | 74 | 71.84 | 82.63 |
| Trimethoprim | 261 | 81.06 | 61 | 73.49 | | 69 | 66.99 | 73.85 |
| Erythromycin | 245 | 76.09 | 72 | 86.75 | | 81 | 78.64 | 80.49 |
| Tetracycline | 271 | 84.16 | 79 | 95.18 | | 77 | 74.76 | 84.70 |
| Doxycycline | 194 | 60.25 | 64 | 77.11 | | 60 | 58.25 | 65.20 |
| Cefotaxime | 279 | 86.65 | 67 | 80.72 | | 61 | 59.22 | 75.53 |
| Cefazolin | 284 | 88.20 | 74 | 89.16 | | 76 | 73.79 | 83.71 |
| Ciprofloxacin | 259 | 80.43 | 60 | 72.29 | | 53 | 51.46 | 68.06 |
| Enrofloxacin | 219 | 68.01 | 47 | 56.63 | | 38 | 36.89 | 53.84 |
| Ofloxacin | 80 | 24.84 | 20 | 24.10 | | 24 | 23.30 | 24.08 |
| Gentamicin | 129 | 40.06 | 26 | 31.33 | | 32 | 31.07 | 34.15 |
| Streptomycin | 263 | 81.68 | 66 | | 79.52 | 64 | 62.14 | 74.44 |

**Table SI 7** Analysis of AMR patterns of *Escherichia coli* isolates from different parts of Cobb broilers by logical regression

|  | Feces | | Feed trough | |
| --- | --- | --- | --- | --- |
| Antimicrobial | OR value | 95% CI | OR value | 95% CI |
| Amoxicillin | 1.432 | 0.311–6.591 | 0.329 | (0.135–0.799)* |
| Ampicillin | 3.450 | 0.445–26.757 | 0.352 | (0.153–0.812)* |
| Florfenicol | 3.126 | (1.090–8.965)* | 0.469 | (0.271–0.810)* |
| Chloramphenicol | 1.996 | 0.870–4.581 | 0.469 | (0.278–0.793)* |
| Trimethoprim | 0.648 | 0.370–1.136 | 0.474 | (0.289–0.779)* |
| Erythromycin | 2.057 | (1.038–4.078)* | 1.157 | 0.677–1.978 |
| Doxycycline | 2.222 | (1.271–3.885)* | 0.921 | 0.587–1.445 |
| Tetracycline | 3.717 | (1.303–10.601)* | 0.557 | (0.326–0.952)* |
| Cefotaxime | 0.645 | 0.343–1.215 | 0.224 | (0.135–0.372)* * |
| Cefazolin | 1.100 | 0.509–2.377 | 0.377 | (0.216–0.656)* |
| Ciprofloxacin | 0.635 | 0.365–1.104 | 0.258 | (0.160–0.414)* * |
| Enrofloxacin | 0.614 | 0.375–1.006 | 0.499 | (0.317–0.783)* |
| Ofloxacin | 0.648 | 0.370–1.136 | 0.474 | (0.289–0.779)* |
| Gentamicin | 0.682 | 0.408–1.142 | 0.674 | 0.420–1.082 |
| Streptomycin | 0.871 | 0.476–1.592 | 0.368 | (0.226–0.600)* * |

Note: The OR denotes the probability of *E. coli* resistance in swabs from feces and feed trough relative to anal, 95 % CI, *p < 0.05，**p < 0.001

**Table SI 8** Distribution of *Escherichia coli* strains resistant to different antibiotics at different ages

| Number of strains resistant | 1 d | %  (n = 150) | 15 d | %  (n = 148) | 26 d | %  (n = 120) | 38 d | % (n = 90) |
| --- | --- | --- | --- | --- | --- | --- | --- | --- |
| 0 | 1 | 0.67 | 0 | 0.00 | 0 | 0.00 | 0 | 0.00 |
| 1 | 1 | 0.67 | 1 | 0.68 | 0 | 0.00 | 0 | 0.00 |
| 2 | 1 | 0.67 | 0 | 0.00 | 2 | 1.67 | 1 | 1.11 |
| 3 | 9 | 6.00 | 0 | 0.00 | 2 | 1.67 | 0 | 0.00 |
| 4 | 7 | 4.67 | 0 | 0.00 | 4 | 3.33 | 1 | 1.11 |
| 5 | 2 | 1.33 | 0 | 0.00 | 1 | 0.83 | 0 | 0.00 |
| 6 | 8 | 5.33 | 1 | 0.68 | 0 | 0.00 | 2 | 2.22 |
| 7 | 12 | 8.00 | 2 | 1.35 | 1 | 0.83 | 5 | 5.56 |
| 8 | 19 | 12.67 | 1 | 0.68 | 2 | 1.67 | 9 | 10.00 |
| 9 | 13 | 8.67 | 3 | 2.03 | 3 | 2.50 | 5 | 5.56 |
| 10 | 12 | 8.00 | 9 | 6.08 | 13 | 10.83 | 7 | 7.78 |
| 11 | 20 | 13.33 | 14 | 9.46 | 20 | 16.67 | 16 | 17.78 |
| 12 | 23 | 15.33 | 25 | 16.89 | 25 | 20.83 | 18 | 20.00 |
| 13 | 13 | 8.67 | 39 | 26.35 | 25 | 20.83 | 17 | 18.89 |
| 14 | 8 | 5.33 | 31 | 20.95 | 17 | 14.17 | 8 | 8.89 |
| 15 | 1 | 0.67 | 22 | 14.86 | 5 | 4.17 | 1 | 1.11 |

**Table SI 9** Distribution of antibiotic resistant *Escherichia coli* isolates from Southern and Northern regions of Cobb broilers.

| Number of strains resistant | Southern region  Number (%) of strains | % (n = 288) | | Northern region  Number (%) of strains | | % (n = 220) | |
| --- | --- | --- | --- | --- | --- | --- | --- |
| 0 | 0 | 0.00 | | 1 | | 0.45 | |
| 1 | 0 | 0.00 | | 2 | | 0.91 | |
| 2 | 1 | 0.35 | | 3 | | 1.36 | |
| 3 | 3 | 1.04 | | 8 | | 3.64 | |
| 4 | 6 | 2.08 | | 6 | | 2.73 | |
| 5 | 1 | 0.35 | | 2 | | 0.91 | |
| 6 | 6 | 2.08 | | 5 | | 2.27 | |
| 7 | 11 | 3.82 | | 9 | | 4.09 | |
| 8 | 20 | 6.94 | | 11 | | 5.00 | |
| 9 | 16 | 5.56 | | 8 | | 3.64 | |
| 10 | 29 | 10.07 | | 12 | | 5.45 | |
| 11 | 42 | 14.58 | | 28 | | 12.73 | |
| 12 | 60 | 20.83 | | 31 | | 14.09 | |
| 13 | 51 | | 17.71 | | 43 | 19.55 | |
| 14 | 32 | | 11.11 | | 32 | | 14.55 |
| 15 | 10 | | 3.47 | | 19 | | 8.64 |

**Table SI 10** Distribution of antibiotic resistant *Escherichia coli* isolates from different parts of Cobb broilers.

|  | Multi-drug resistance | | | | | |
| --- | --- | --- | --- | --- | --- | --- |
| Number of strains resistant | Anal | % (n = 322) | Feed trough | % (n = 103) | Feces | % (n = 83) |
| 0 | 0 | 0.00 | 1 | 0.97 | 0 | 0.00 |
| 1 | 0 | 0.00 | 2 | 1.94 | 0 | 0.00 |
| 2 | 0 | 0.00 | 4 | 3.88 | 0 | 0.00 |
| 3 | 7 | 2.17 | 4 | 3.88 | 0 | 0.00 |
| 4 | 2 | 0.62 | 9 | 8.74 | 1 | 1.20 |
| 5 | 0 | 0.00 | 3 | 2.91 | 0 | 0.00 |
| 6 | 9 | 2.80 | 1 | 0.97 | 1 | 1.20 |
| 7 | 12 | 3.73 | 6 | 5.83 | 2 | 2.41 |
| 8 | 21 | 6.52 | 7 | 6.80 | 3 | 3.61 |
| 9 | 10 | 3.11 | 8 | 7.77 | 6 | 7.23 |
| 10 | 24 | 7.45 | 8 | 7.77 | 9 | 10.84 |
| 11 | 47 | 14.60 | 7 | 6.80 | 16 | 19.28 |
| 12 | 67 | 20.81 | 11 | 10.68 | 13 | 15.66 |
| 13 | 57 | 17.70 | 16 | 15.53 | 21 | 25.30 |
| 14 | 42 | 13.04 | 12 | 11.65 | 10 | 12.05 |
| 15 | 24 | 7.45 | 4 | 3.88 | 1 | 1.20 |
